# Efficacy of sonothrombolysis as an adjunct to primary percutaneous coronary intervention in ST-segment elevation myocardial infarction: a systematic review and meta-analysis

**DOI:** 10.1007/s11239-025-03176-1

**Published:** 2025-09-09

**Authors:** Mohamed Abo Zeid, Ahmed Farid Gadelmawla, Kareem Khalefa, Ahmed Yasser Shaban

**Affiliations:** 1 Faculty of Medicine, Tanta University, Tanta, Egypt; 2 Faculty of Medicine, Menoufia University, Menoufia, Egypt; 3 Faculty of Medicine, Kafrelsheikh University, Kafrelsheikh, Egypt

**Keywords:** Sonothrombolysis, Ultrasound-enhanced thrombolysis, pPCI, Myocardial infarction, STEMI, Myocardial ischemia

## Abstract

In this review, we aimed to evaluate Sonothrombolysis when combined with primary percutaneous coronary intervention (pPCI) in STEMI patients with regard to improving cardiac function and clinical outcomes. This study primarily assesses short-term efficacy outcomes, while long-term impacts, such as mortality, were not evaluated. Following the PRISMA (Preferred Reporting Items for Systematic Reviews and Meta-Analyses) guidelines, we searched four electronic databases (PubMed, Scopus, Cochrane Library, and Web of Science) to identify eligible studies reported up to November 2024. Four studies, with a total population of 252 participants, were included. The sonothrombolysis group demonstrated an overall greater improvement in left ventricular ejection fraction compared to the control group (MD = 3.07, 95% CI [1.20 to 4.94], *p* = 0.001), with no heterogeneity (*p* = 0.44, I^2^ = 0%). When subgrouped according to the follow-up period, there was no significant difference between the two groups (MD = 2.56, 95% CI [-0.35 to 5.46]) after 2 to 6 months. Infarction size, microvascular obstruction, left ventricular end-diastolic volume, and left ventricular end-systolic volume showed no statistically significant difference between the two groups. Sonothrombolysis following pPCI is associated with better left ventricular ejection fraction, emphasizing the potential role of sonothrombolysis as an adjunctive therapy to pPCI in the management of STEMI.

**Graphical abstract:**

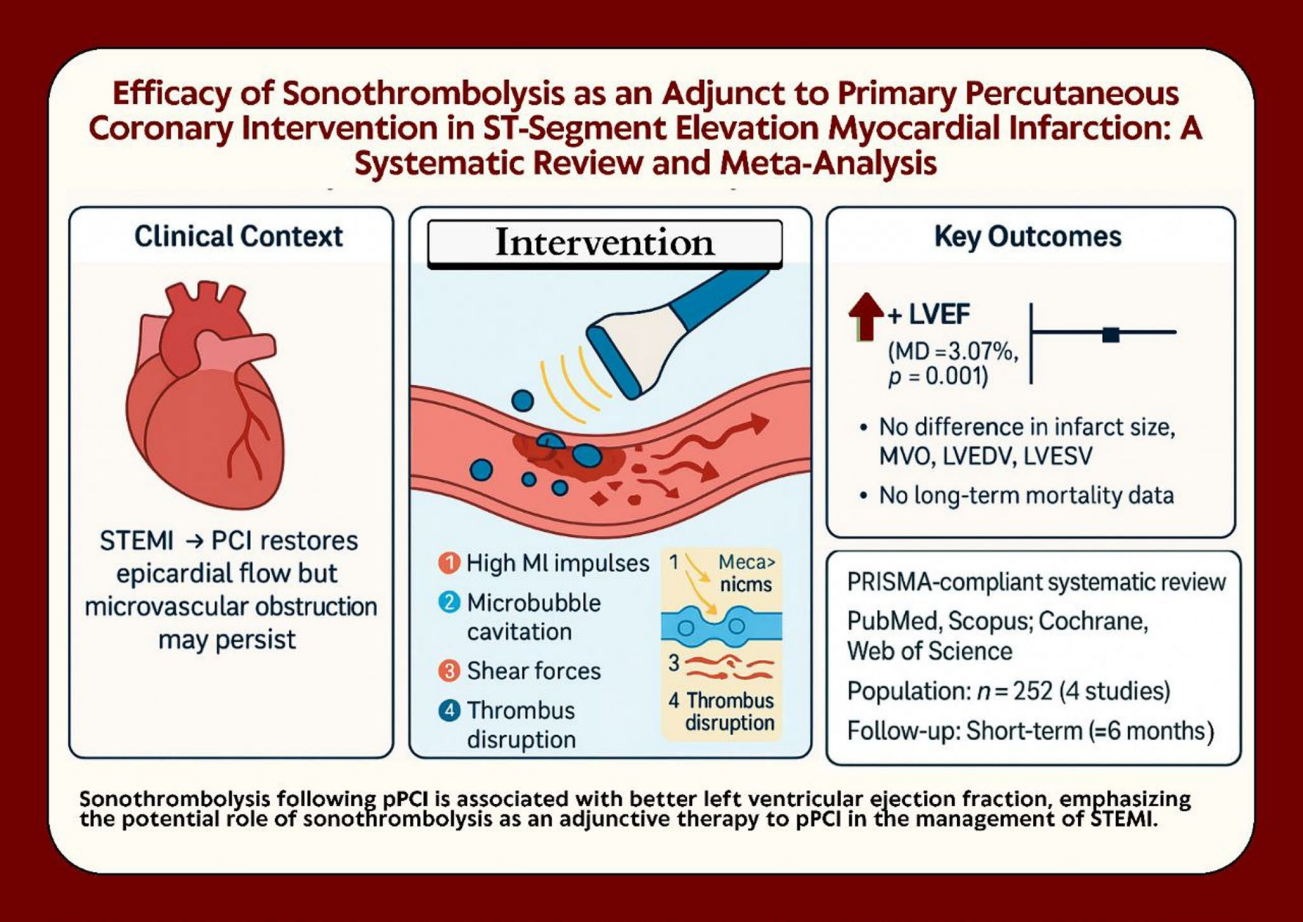

**Supplementary Information:**

The online version contains supplementary material available at 10.1007/s11239-025-03176-1.

## Introduction

Acute coronary syndrome (ACS) includes conditions that involve a rapid reduction in blood flow to the heart muscle, manifesting as ST-segment elevation myocardial infarction (STEMI) due to the complete obstruction of a coronary artery, non-ST-segment elevation myocardial infarction (NSTEMI), or unstable angina [1]. The standard treatment is primary percutaneous coronary intervention (pPCI). While PCI can reopen the obstructed epicardial coronary artery responsible for the infarction, many patients still do not achieve optimal microvascular perfusion. This phenomenon, known as “no-reflow,” occurs due to microvascular obstruction (MVO) that persists even after blood flow is restored in the epicardial coronary artery following PCI [2–5]. There is a need for supplementary cardioprotective strategies to preserve myocardial function in STEMI patients [6]. 

The use of diagnostic ultrasound to evaluate microvascular perfusion with ultrasound enhancing agents is considered a standard practice after emergency PCI treatment for STEMI. This assessment is conducted using low mechanical index (MI) imaging to analyze regional wall motion and microvascular blood volume, along with intermittent high MI impulses to assess myocardial microvascular blood flow. It has been revealed that the repeated application of diagnostic high MI impulses during the infusion of contrast microbubbles can effectively restore flow in cases of acute STEMI [7–10]a process referred to as Sonothrombolysis (STL). The cavitation of microbubbles caused by high MI impulses generates shear forces that help dissolve thrombi in both the coronary arteries and microvasculature [8, 9, 11, 12]. STL can help restore both epicardial and microvascular blood flow in cases of acute STEMI [8]. A recent clinical trial conducted at a single center found that using STL before and after primary PCI enhanced the patency of the infarct-related arteries, improved microvascular circulation, reduced the size of the infarction, and led to better ejection fraction outcomes after six months; however, optimal microvascular perfusion is not always achieved due to the no-reflow phenomenon [13]. 

Although several randomized controlled trials have examined the effectiveness of STL, the majority have focused on its application in stroke [14]. Findings have been inconsistent, and its potential role in myocardial infarction has not been thoroughly investigated. This study aims to evaluate the efficacy of STL as an adjunct to primary PCI in patients with STEMI by assessing angiographic outcomes, microvascular perfusion, infarct size, and left ventricular (LV) systolic function. We hypothesize that STL may reduce infarct size and improve systolic function by enhancing microvascular reperfusion.

## Methods

This study was reported according to the Preferred Reporting Items for Systematic Reviews and Meta-Analyses (PRISMA) guidelines [15, 16]. In addition, this review protocol was registered with Open Science Framework (OSF) (DOI: 10.17605/OSF.IO/M4WSZ).

### Search strategy

We searched PubMed, Scopus, Web of Science, and Cochrane Library databases up to November 2024 using the search strategy mentioned in Supplementary Table 1.

Duplicates were removed using Endnote software (Clarivate Analytics, PA, USA). The retrieved references were uploaded to the Rayyan website [17] and screened in two steps: the first consisted of screening the titles/abstracts independently by (A. Y. S., A. F.) to determine their relevance, and the second consisted of screening the full-text articles. The studies were retrieved for a full-text check to assess their eligibility against the criteria. The full text of all related articles was then obtained and checked by at least two independent authors (A. Y. S., S. F.). The disagreements were resolved by a senior author’s decision (M. A.)

### Selection of studies

We included studies that met these inclusion criteria: 1- Studies enrolled patients undergoing STL combined with PCI versus PCI alone or with sham procedures. 2- RCTs reporting efficacy and safety outcomes were included in our meta-analysis. 3- Studies published in international peer-reviewed journals. We excluded the previous observational studies, reviews, preclinical studies, animal studies, pharmacokinetics, and pharmacodynamics studies, lacking clear clinical outcomes. Any disagreement in study selection was solved by consultation with a senior reviewer, and final decisions were based on consensus.

### Data extraction and risk of bias assessment

Two authors independently extracted the data (M. A. and K. K.) using an online Google sheet form. Any conflicts were resolved by a third reviewer (A. F. G.). Data was extracted across three domains: 1: Study characteristics (study ID, study design, country, total participants, follow-up duration, main inclusion criteria and the number of applied flashes/impulses). 2: Characteristics of the included study population including age, sex, BMI, and comorbidities (diabetes mellitus, hypertension, and hyperlipidemia). 3: Study outcomes including angiographic characteristics (thrombus aspiration, predilatation, postdilatation, and Infarct territory), MRI findings after two to three days and two to six months (infarction size, relative Infarction size, left ventricular ejection fraction, MVO, left ventricular end-diastolic volume (EDV), left ventricular end-systolic volume (ESV), and left ventricular mass), and echo finding (left ventricular ejection fraction (LVEF) after two to three days and two to six months).

Infarct size was defined as the proportion of left ventricular (LV) myocardial mass that is irreversibly damaged, while MVO was defined as the extent of hypo enhanced zones within the infarcted myocardium on early or late gadolinium enhancement images. Both are quantified in grams or expressed as a percentage of total LV mass using cardiac magnetic resonance imaging (CMR).

For risk of bias assessment, two independent authors reviewed each study’s protocol and full text, as well as any supplementary materials available. We utilized the revised Cochrane risk-of-bias tool for RCTs (RoB 2) to evaluate the risk of bias in the included clinical trial [18]. This evaluation encompassed an assessment of the randomization process, concealment of the allocation sequence, deviations from the intended interventions, utilization of appropriate analysis to estimate the effect of assignment to intervention, measurement of the outcome, selection of the reported results, and overall risk of bias. The assessment of the methodological quality of the studies was classified as either low risk, with some concerns, or high risk of bias.

### Statistical analysis

We used RevMan v5.4.1 to conduct statistical analysis [19]. Continuous variables were presented as the mean difference (MD) and the corresponding 95% confidence intervals (CI). Random-effect model was used in case of significant heterogeneity. I-square (I^2^) and Chi-square (X^2^) tests were used to evaluate heterogeneity, where the X^2^ test detects the presence of heterogeneity, and the I^2^ test evaluates its degree. We interpreted I² according to Cochrane guidelines [16]: 0–40% (low), 30–60% (moderate), 50–90% (substantial), 75–100% (considerable) with a significance level below 0.1 in the X2 test indicated significant heterogeneity. We used leave-one-out sensitivity analysis and subgrouping by follow-up duration (short-term: 2–3 days; intermediate-term: 2–6 months) to explore heterogeneity when feasible. Missing SDs were calculated from SEs or 95% CIs per Altman’s method [20]. 

## Results

### Search results and study selection

**Fig. 1:**
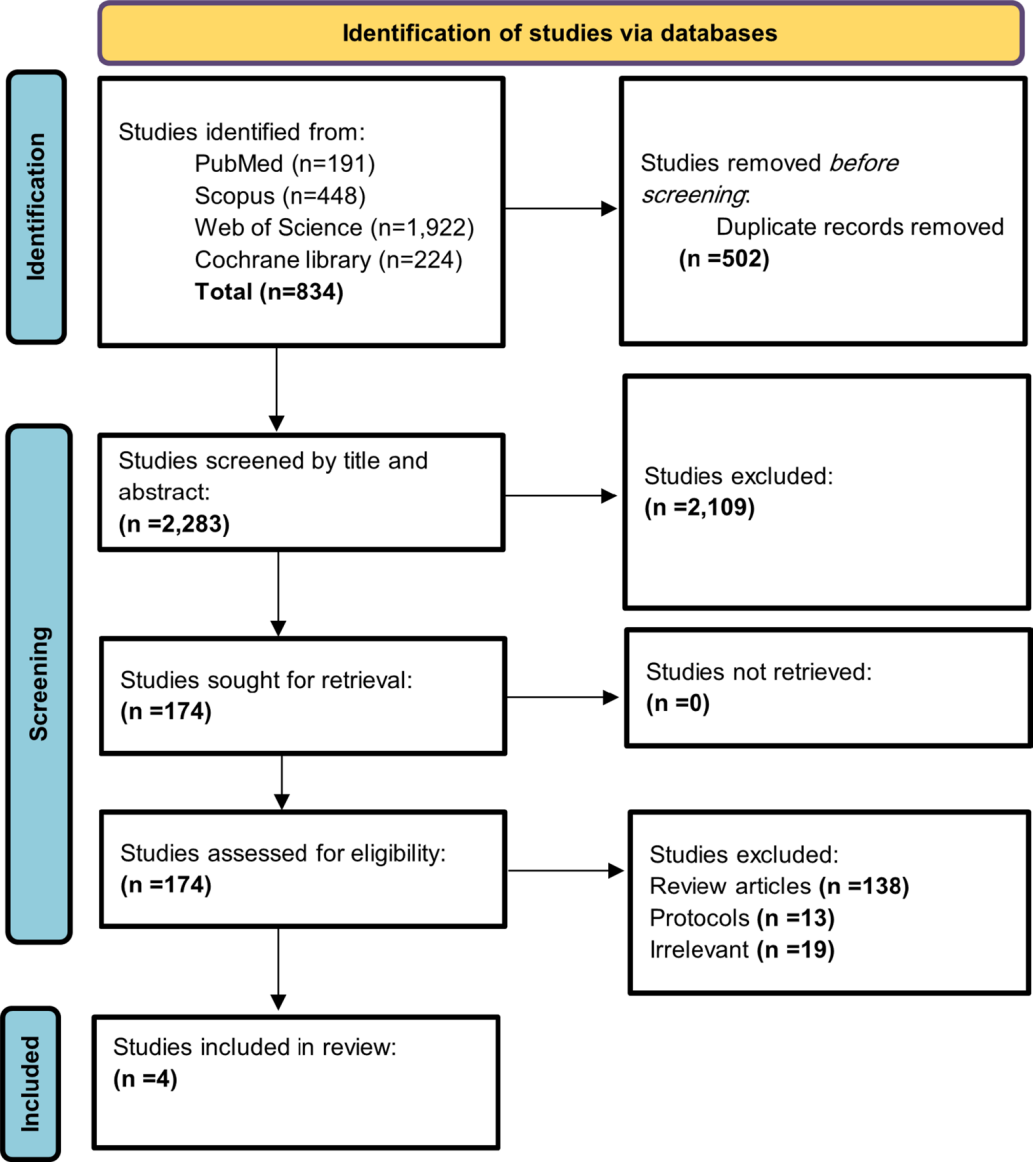
PRISMA flowchart

Following our systematic search, 2,785 papers were obtained. After removing 502 duplicates, the remaining 2,283 studies went through the title and abstract screening leading to the exclusion of 2,109 studies that did not meet our eligibility criteria. Then we screened the full texts of 174 studies, identifying four studies that met our eligibility criteria (Fig. 1).

### Characteristics of the included studies

Four RCTs were included in this meta-analysis [13, 21–23]comprising a total of 252 patients, 129 of whom received PCI followed by STL (the intervention group) and the remaining 123 received PCI alone or with very low MI to detect regional wall motion and microvascular perfusion (the control group) Table 1. The mean age of the patients ranged between 66 ± 13 to 57.5 ± 8.5 years. The study population included 56 (22.2%) diabetic patients, 90 (35.7%) with hyperlipidemia, 117 (46.4%) with hypertension, 90 (35.7%) smokers, and 12 (4.8%) with a history of previous PCI. Furthermore, the study population received various medications like aspirin, beta blockers, calcium channel blockers, statins, ACEIs and ARBs. These medications may have introduced confounding and contributed to inconsistencies and heterogeneity. For further elaboration of patients’ demographics and medical history see Table 2.

**Table 1:** Summary of the included studies

Study ID	Study design	Study arms		Country	Total participants	Follow-up duration	Main inclusion criteria	Number of applied flashes/impulses
		Intervention	Control					
El Kadi 2024	Randomized controlled trial	PCI followed by sonothrombolysis in the form of Very low and high MI	PCI followed by very low MI	The Netherlands	67	6-month	Age > 30 years - Acute (within 12 h) or worsening chest pain or shortness of breath associated with: o ≥ 2 mm ST-segment elevation in 2 anterior or lateral leads; or o ≥ 1 mm ST-segment elevation in 2 inferior leads o ≥ 1 mm ST-segment elevation in lateral leads (I, aVL, V5, V6) - Persistence of ST-elevation of equal to or more than 30% in the lead with the highest ST-elevation on the ECG after PCI compared to the ECG before PCI - Adequate echocardiographic images	60–90 flashes in total
Jeyaprakash 2024	pilot study	Sham pre and sonothrombolysis post PCI	sham pre-/post- PCI	Australia	35	6 months	if they presented with STEMI for the first time were between 30 and 80 years of age, were suitable for pPCI.	30 flashes
Li 2024	randomized controlled trial	Sonothrombolysis (low MI imaging with intermittent high MI impulses) following the PCI	low MI alone following the PCI	The Netherlands	50	6 months	(1) Age > 30 years; (2) Acute (within 12 h) or worsening chest pain or short of breath (SOB) associated with > 1.0 mV ST elevation on Electrocardiogram (EKG) in at least two contiguous anterior precordial leads; (3) Willing to undergo coronary angiography within 12 h of presentation.; (4) Adequate echocardiographic images; (5) Successful PCI with patent vessel and at least Thrombolytics in Myocardial Infarction (TIMI) 2 grade flow in the LAD post-PCI	30 impulses
Mathias 2019	Randomized controlled trial	PCI followed by Sonothrombolysis (low and high MI)	PCI only followed by low MI	Brazil	100	6 months	Age ≥ 30 years with STEMI with less than 12 h of chest pain onset. Eligible for emergent PCI therapy. No contraindications or hypersensitivities to ultrasound contrast agents	60–90 impulses

PCI: Percutaneous coronary intervention; MI: Mechanical index; ECG: Electrocardiography; TIMI: Thrombolysis in Myocardial Infarction; LAD: Left anterior descending; STEMI: ST-segment elevation myocardial infarction

**Table 2:** Demographics and baseline characteristics of patients in the included studies. This table presents demographic differences among studies; for example, Li 2024 reported a higher percentage of smokers in the intervention group (52%) while Mathias 2024 a higher percentage of DM in the intervention group (42%)

Study ID	Study arms	Age (years), mean (SD)	BMI (kg/m2), mean (SD)	Sex (female), N (%)	DM, N (%)	Hypertension, N (%)	Hyperlipidemia, N (%)	Smoking, N (%)	Previous PCI, N (%)	Positive family history, N (%)	Drugs used before admission, N (%)				
											Aspirin	Statin	Beta-blocker	Calcium-channel blocker	ACE-i/ARB
El Kadi 2024	Sonothrombolysis	65 (13)	26 (3.09)	1 (3%)	8 (23%)	6 (17%)	10 (30%)	6 (17%)	3 (9%)	7 (20)	3 (9%)	4 (11%)	1 (3%)	3 (9%)	3 (9%)
	Control	66 (13)	26 (3.10)	8 (25%)	2 (6%)	12 (38%)	6 (19%)	8 (25%)	3(9%)	5 (16)	4 (13%)	3 (9%)	3 (9%)	1 (3%)	5 (16%)
Jeyaprakash 2024	Sonothrombolysis	61.67 (8.81)	29.97 (6.41)	3 (16%)	2 (11%)	7 (37%)	8 (42%)	7 (37%)	NA	NA	NA	NA	NA	NA	NA
	Control	59 (10.57)	29.63 (3.82)	3 (19%)	3 (19%)	9 (56%)	9 (56%)	5 (31%)	NA	NA	NA	NA	NA	NA	NA
Li 2024	Sonothrombolysis	57.5 (8.5)	29.3 (3.9)	6 (24%)	5 (20%)	12 (48%)	10 (40%)	13 (52%)	2 (8%)	8 (32%)	6 (24%)	7 (28%)	4 (16%)	5 (20%)	5 (20%)
	Control	59.1 (10.5)	29.3 (4.8)	4 (16%)	4 (16%)	15 (60%)	12 (48%)	7 (28%)	4 (16%)	10 (40%)	5 (20%)	5 (20%)	5 (20%)	5 (20%)	9 (36%)
Mathias 2019	Sonothrombolysis	59 (10)	NA	NA	21 (42%)	28 (56%)	20 (40%)	24 (48%)	NA	NA	48 (96%)	19 (38%)	14 (28%)	5 (10%)	NA
	Control	59 (11)	NA	NA	11 (22%)	28 (56%)	15 (30%)	20 (40%)	NA	NA	50 (100%)	14 (28%)	5 (10%)	4 (8%)	NA

DM: Diabetes mellitus; ACE-I: Angiotensin-Converting Enzyme Inhibitors; ARBs: Angiotensin II Receptor Antagonists

In the STL group 87 patients had their left anterior descending artery (LAD) occluded, 31 had their right coronary artery (RCA) occluded and 11 had their left circumflex artery (LCx) occluded, while in the control group 80 patients had their LDA occluded, 29 had their RCA occluded and 14 had their LCx occluded as demonstrated in Table 3.

**Table 3:** Baseline angiographic findings of patients in the included studies. This table highlights that 49% of patients in the sonothrombolysis group had predilatation, with 77% showing LAD involvement, while the control group had 38% predilatation and 69% LAD involvement. TIMI flow grade 2 was observed in 63% of the sonothrombolysis group versus 72% in the control group

Study ID	Study arms	Thrombus aspiration, N (%)	Predilatation, N (%)	Postdilation, N (%)	IIb/IIIa inhibitor use, N (%)	Infarct territory, N (%)			TIMI flow grade before PCI, N (%)			
						LAD	LCx	RCA	0	1	2	3
El Kadi 2024	Sonothrombolysis	0	17 (49)	9 (26)	0	27 (77)	2 (6)	6 (17)	22 (63)	6 (17)	5 (14)	2 (6)
	Control	0	12 (38)	5 (16)	0	22 (69)	2 (6)	8 (25)	23 (72)	2 (6)	6 (19)	1 (3)
Jeyaprakash 2024	Sonothrombolysis	6 (32)	17 (89)	16 (84)	5 (26)	9 (47)	2 (11)	8 (42)	10		9	
	Control	6 (38)	11 (69)	11 (69)	5 (31)	7 (44)	2 (12)	7(44)	12		4	
Li 2024	Sonothrombolysis	4 (16)	24 (96)	21 (84)	3 (12)	25 (100)	0	0	13 (52)	1 (4)	4 (16)	4 (16)
	Control	5 (20)	25 (100)	20 (80)	5 (20)	25 (100)	0	0	15 (60)	2 (8)	7 (28)	4 (36)
Mathias 2019	Sonothrombolysis	0	NA	NA	NA	26 (52)	7 (14)	17 (34)	22 (44)	4 (8)	8 (16)	16 (32)
	Control	1 (2)	NA	NA	NA	26 (52)	10 (20)	14 (28)	35 (70)	5 (10)	3 (6)	7 (14)

PCI: Percutaneous coronary intervention; TIMI: Thrombolysis in Myocardial Infarction; LAD: Left anterior descending; LCx: Left Circumflex Artery; RCA: Right coronary artery

### Risk of bias and quality of evidence

As seen in Supplemental Figs. 1 and 2, all studies showed low risk of bias in all domains except for Mathias et al. [13] showed an overall “some concerns,” mainly in the randomization domain.

### Efficacy outcomes

#### Left ventricular ejection fraction

**Fig. 2:**
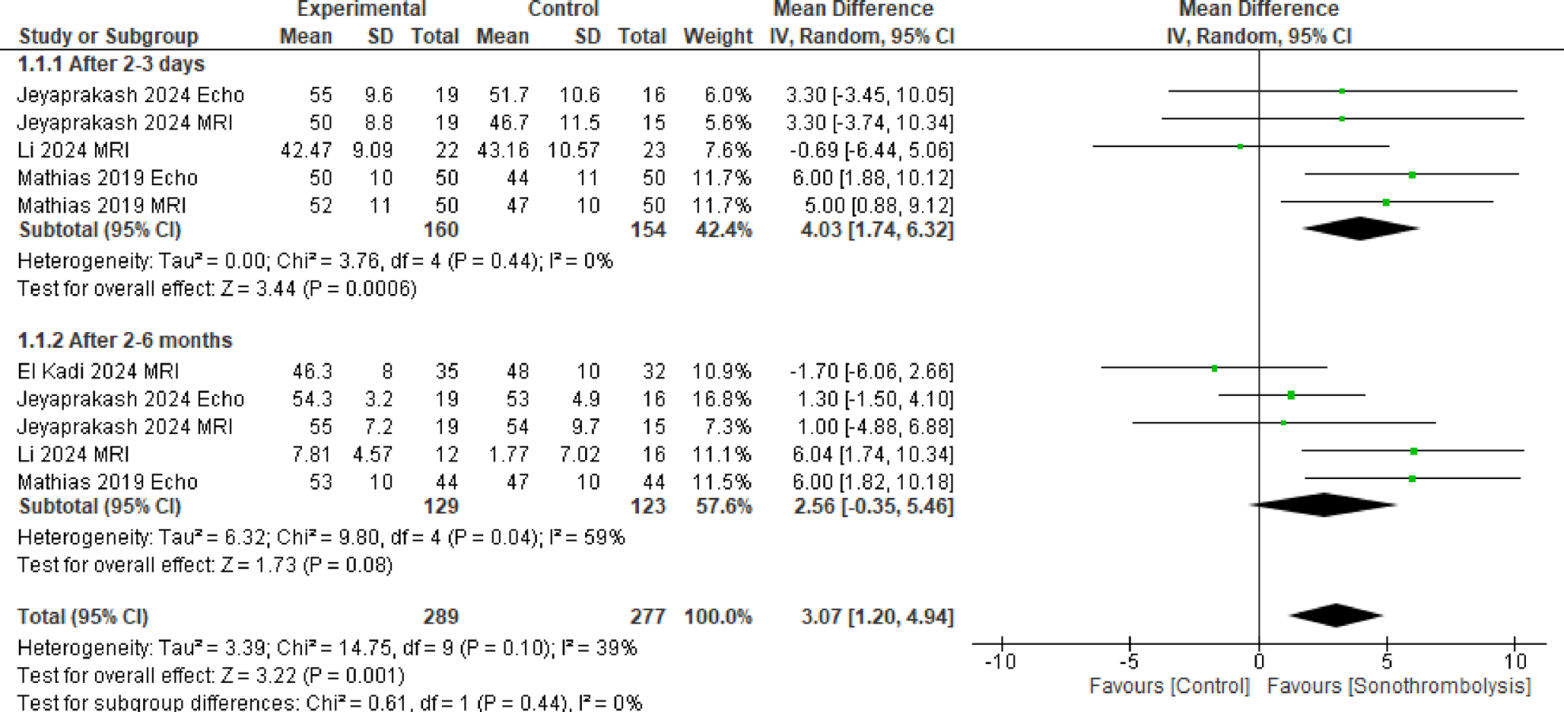
Left ventricular ejection fraction. The intervention group had a statistically significant improvement in left ventricular ejection fraction (p < 0.05)

All four studies were included in this pooled result with the overall mean difference between the two groups favoring the STL group (MD = 3.07, 95% CI [1.20 to 4.94], *p* = 0.001), with no significant heterogeneity (*p* = 0.10, I^2^ = 39%) (Fig. 2).

When analyzed after 2 to 3 days, 91 patients were included in the intervention group and 88 in the control group and we found that the STL group showed significantly higher results (MD = 4.03, 95% CI [1.74 to 6.32]), with no significant heterogeneity (*p* = 0.44, I^2^ = 0%), but when evaluated after 2 to 6 months, 91 patients were included in the intervention group and 64 in the control group and we found that there was no significant difference between the two groups (MD = 2.56, 95% CI [−0.35 to 5.46]), with significant heterogeneity (*p* = 0.04, I^2^ = 59%). To deal with heterogeneity a leave-one-out sensitivity analysis was performed, and it was better resolved by excluding El Kadi et al. [21] showing statistically significant results favoring the intervention group after 2 to 6 months (MD = 3.55, 95% CI [0.72 to 6.38], *p* = 0.01). Supplemental Fig. 3.

#### Infarction size

**Fig. 3:**
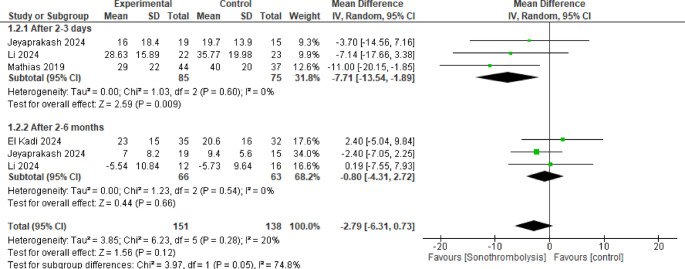
Infarction size. The intervention group had a statistically significant improvement in infarction size after 2-3 days (p < 0.05), but not after 2-6 months (p >0.05)

All four studies were included in this pooled result with the overall mean difference between the two groups not favoring either of them (MD = −2.79, 95% CI [−6.31 to 0.73], *p* = 0.12), with no significant heterogeneity among the pooled evidence (*p* = 0.28, I^2^ = 20) (Fig. 3).

When analyzed after 2 to 3 days, 85 patients were included in the intervention group and 75 in the control group and we found that the STL group showed significantly lower results (MD = −7.71, 95% CI [−13.54 to −1.89]), with no significant heterogeneity (*p* = 0.60, I^2^ = 0%), but when evaluated after 2 to 6 months, 66 patients were included in the intervention group and 63 in the control group and there was no significant difference between the two groups (MD = −0.80, 95% CI [−4.31 to 2.72]), with no significant heterogeneity (*p* = 0.28, I^2^ = 0%). But significant heterogeneity was found between the subgroups (*p* = 0.05, I^2^ = 74.8%) and it was better resolved by excluding Mathias et al. [13] showing no statistically significant difference between the two groups after 2 to 3 days (MD = −5.47, 95% CI [−13.03 to 2.08], *p* = 0.16). Supplemental Fig. 4.

#### Relative infarction size

**Fig. 4:**
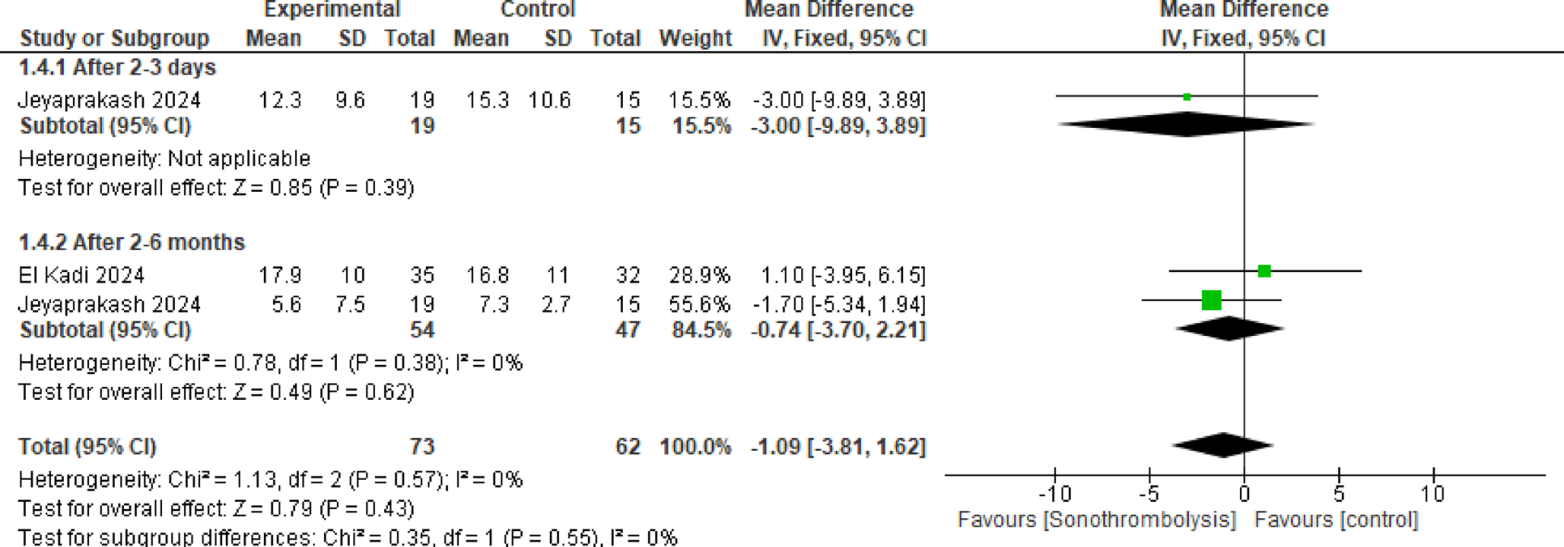
Relative Infarction size. No statistically significant difference was observed between the two interventions in either subgroup (p >0.05)

Across the two studies included in this pooled result, 54 patients were included in the STL group, and 47 in the control group and no statistically significant difference was observed between the two groups after 2 to 3 days and 2 to 6 months (MD = −3.00, 95% CI [−9.89 to 3.89], *p* = 0.39) and (MD = −0.74, 95% CI [−3.70 to 2.21], *p* = 0.62) respectively (Fig. 4).

#### Microvascular obstruction

**Fig. 5:**
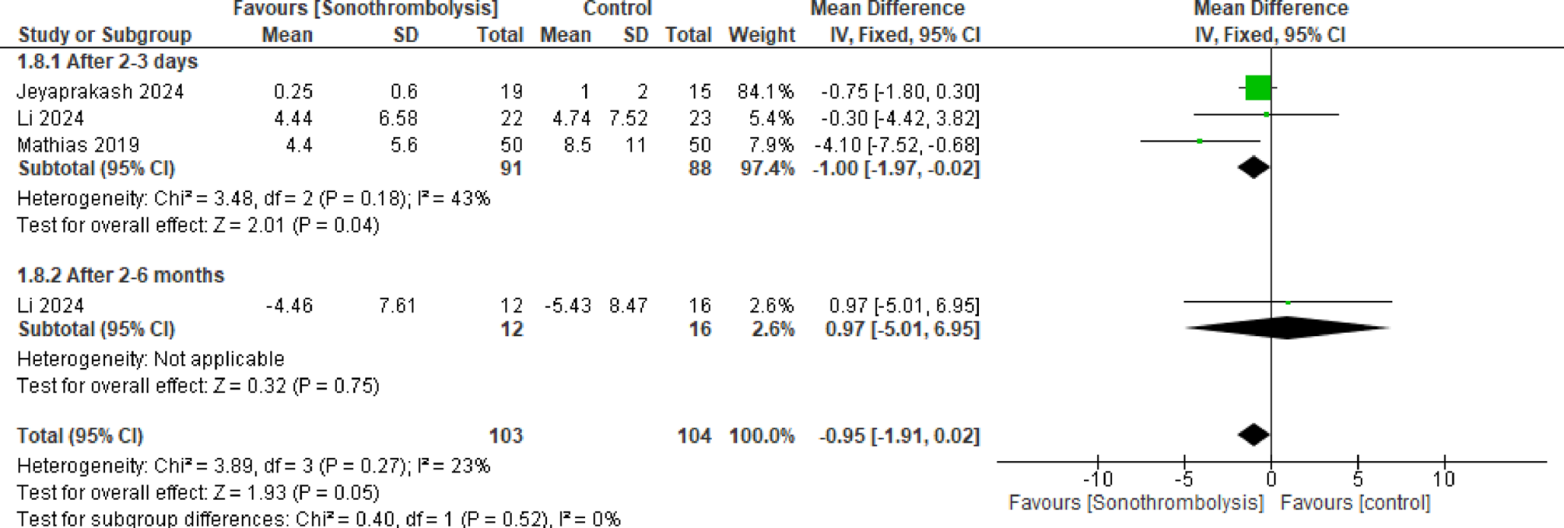
Microvascular obstruction. The intervention group had a statistically significant improvement in infarction size after 2-3 days (p < 0.05), but not after 2-6 months (p >0.05)

Only two studies were included in this pooled result with an overall mean difference not favoring either of the two groups (MD = −0.95, 95% CI [−1.91 to 0.02], *p* = 0.05), with no heterogeneity (*p* = 0.27, I^2^ = 23%) (Fig. 5).

When analyzed after 2 to 3 days, 91 patients were included in the intervention group and 88 in the control group and we found that the STL group demonstrated significantly lower results (MD = −1.00, 95% CI [−1.97 to −0.02]), with no significant heterogeneity (*p* = 0.18, I^2^ = 43%), but when evaluated after 2 to 6 months there was no significant difference between the two groups (MD = 0.97, 95% CI [−5.01 to 6.95]) and the heterogeneity was not estimated.

#### Left ventricular end-diastolic volume

**Fig. 6:**
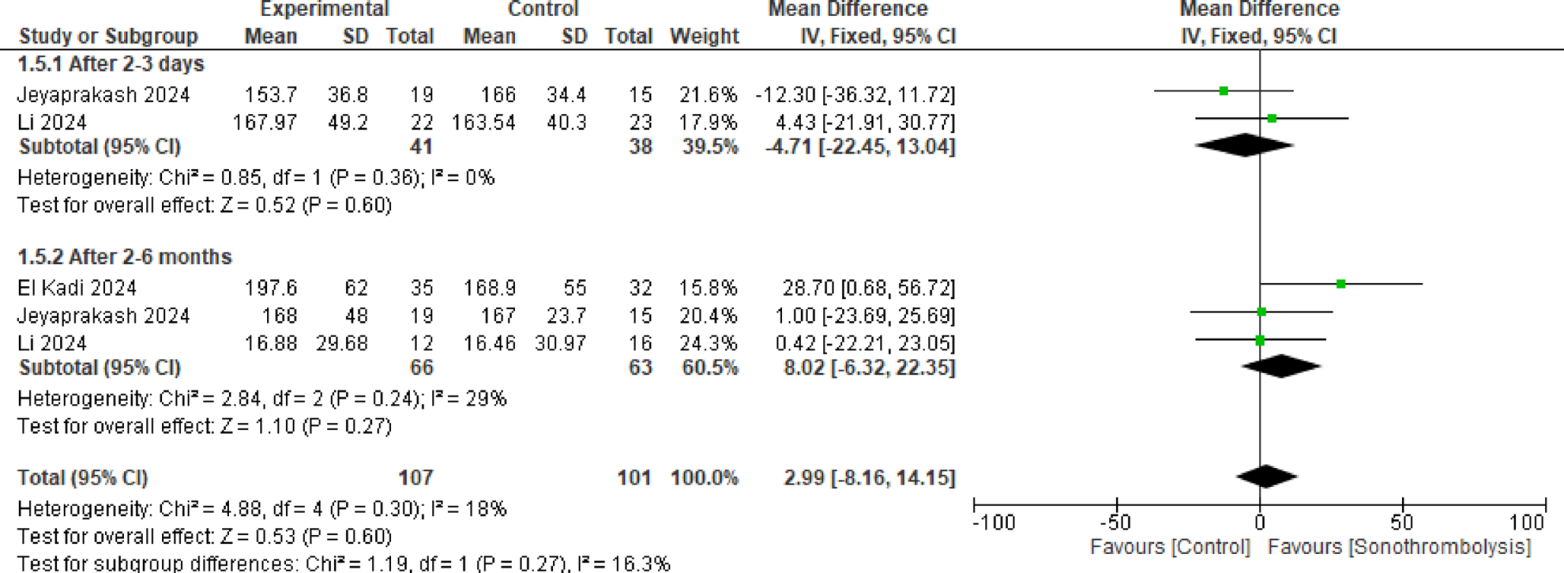
Left ventricular end-diastolic volume. No statistically significant difference was observed between the two interventions in either subgroup (p >0.05)

Three studies were analyzed in this pooled result including 76 patients in STL group and 70 in the control, but no statistically significant difference was observed between the two groups after 2 to 3 days and 2 to 6 months (MD = −4.71, 95% CI [−22.45 to 13.04], *p* = 0.60) and (MD = 8.02, 95% CI [−6.32 to 22.35], *p* = 0.27) respectively with no significant heterogeneity in either or between the subgroups (Fig. 6).

#### Left ventricular end-systolic volume

**Fig. 7:**
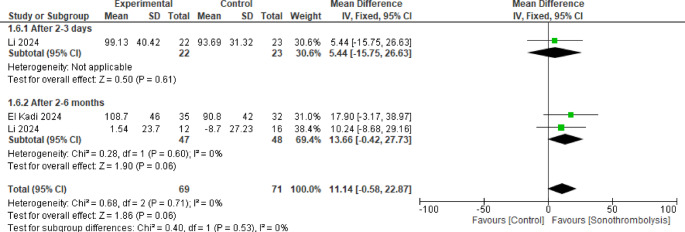
Left ventricular end-systolic volume. No statistically significant difference was observed between the two interventions in either subgroup (p >0.05)

Two studies were analyzed in this pooled result including 47 patients in STL group and 48 in the control with no statistically significant difference between the two groups after 2 to 3 days and 2 to 6 months (MD = 5.44, 95% CI [−15.75 to 26.63], *p* = 0.61) and (MD = 13.66, 95% CI [−0.42 to 27.73], *p* = 0.06) respectively and no significant heterogeneity in either or between the subgroups (Fig. 7).

#### Left ventricular mass

**Fig. 8:**
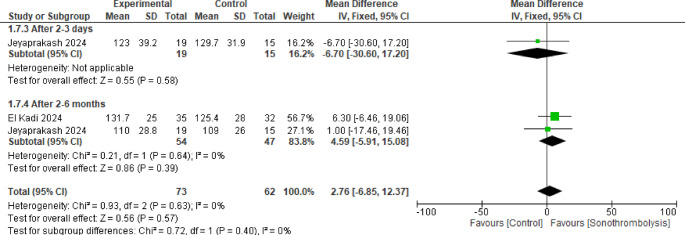
Left ventricular mass. No statistically significant difference was observed between the two interventions in either subgroup (p >0.05)

Two trials were analyzed in this pooled result including 54 patients in STL group and 47 in the control group with no statistically significant difference between the two groups after 2 to 3 days and 2 to 6 months (MD = −6.70, 95% CI [−30.60 to 17.20], *p* = 0.58) and (MD = 4.59, 95% CI [−5.91 to 15.08], *p* = 0.39) respectively and no significant heterogeneity in either or between the subgroups (Fig. 8).

## Discussion

STL has been widely utilized in the treatment of ischemic stroke, and has proven to be a feasible, inexpensive, relatively safe, and a non-invasive technique for treating with stroke patients when compared to standard intravenous thrombolysis [24–27]. However, there is a scarcity in literature considering its use in myocardial infarction [27]. The effect of STL is thought to be primarily stemming from the combined role of the high mechanical index (HMI) radiation pulses and their counterpart, microbubbles (MBs). Microbubbles are gas-filled spheres from contrast agents used during STL. They can be loaded with drugs or even genes encased in lipid or lipopolymer shells [28]. MBs are then capable of inducing a set of shear forces that accelerate thrombus elimination and enhance the activity of fibrinolytic enzymes. Another mechanism through which STL acts is by activation of mechanoreceptors in the wall of the occluded vessels which hence stimulates the release of vasodilators such as nitric oxide (NO) and adenosine [29–32]. Heat generated during application of ultrasound may also contribute to clot disaggregation via thermal effects, according to energy conservation principles. As sound energy becomes absorbed by tissues, it is then converted into heat. However, this mechanism is not definitively accepted as a main mechanism according to previous studies [33, 34]. Although sole application of very low mechanical index (VLMI) ultrasound could be as effective as when combined with MBs if given through prolonged duration, addition of a tissue plasminogen activator (TPA) could significantly improve clot lysis outcome [35]. The efficacy of using STL alone, however, remains largely unclear and hence we proceeded with a review analysis on the topic given the present, regardless of its limitedness, evidence.

A set of seven efficacy outcomes including LVEF, absolute and limited infarction size, MVO, left ventricular EDV and ESV, and left ventricular mass have been respectively examined in contrast to control groups where a sham PCI procedure was applied.

LVEF witnessed an overall mild improvement in the intervention group with no significant heterogeneity noted. It has long been investigated the role of PCI in improving left ventricular function which was later proved to be a strong prognosticator of long-term mortality rate. It is further suggested that overall enhancement of LVEF is controlled by original left ventricular baseline systolic function. Where significant improvements were recorded in patients with more severe dysfunction, PCI almost had no impact on those with preserved baseline systolic function [36]. Similar results were retrieved upon cardiac catheterization [37]. Thus, it is essential to carefully interpret the current results or generalize them broadly given that some underreported factors such as the baseline condition of the heart itself could contribute to significant turnovers in the results especially with the limited literature.

The trending growing interest in applying STL in favor of other more invasive interventions such as PCI is, however, limited by certain restrictions. One of these is persistent ischemia, mostly leading to reperfusion injury upon application of the technique which cannot be prevented by STL [38]. Another is potential post-STL no-reflow phenomenon which has been previously underreported in literature, thus the exact impact of STL on quality of life remains unclear [39–41]. No-reflow phenomenon occurs in up to 14% of patients with presumed successful PCI and share in development of severe complications up to death. The incidence of no-reflow is thought to be underestimated due to the limited utilization of contemporary diagnostic methods. Moreover, it is more likely to occur in patients with predisposing conditions such as hypertension, diabetes mellitus, and obesity [42]. These conditions have already been excessively reported in the included studies, suggesting liability for the phenomenon. Based on the concluded results, MVO with possible manifestation as no-reflow phenomenon showed no significant difference between both the intervention and the control group, however, a more favorable outcome presented in STL group only on short-term basis. This variability between short-term and long-term outcomes could be explained in the light of the number of participants in each subgroup analysis, as well as prior medication use, which may have skewed the results, and the aforementioned difficulty in assessment. Our analysis reported that the STL group was associated with clinical improvement in the outcomes of MVO as well as the absolute and relative infarction size. However, this improvement failed to reach statistical significance in the six-month follow-up period. This lack of significance could be attributed to various factors, including underpowered analysis due to the relatively small sample size, inability and the limited sensitivity of imaging methods such as MRI or echo to detect small and subtle changes in these parameters and associated baseline data of the pooled population that could affect the final outcomes. Notably, a high proportion of patients had STEMI affecting the anterior myocardial wall, which is commonly associated with a higher incidence of the no-reflow phenomenon, which could have influenced the final outcomes [43]. 

Both left ventricular EDV and ESV, as well as left ventricular mass remained widely unchanged upon shifting to STL. PCI has previously been recorded to mitigate diastolic dysfunction and hence significantly improve end-diastolic volume majorly through enhancing previously impaired relaxation. LVEDV is also thought to be in positive correlation with decreased mortality rates [44, 45] Similarly, a favorable decrease in end systolic volume has been previously recorded post PCI suggesting a return to improved cardiac function [46]. Thus, the positive enhancement in the overall cardiac function could be attributed to the use of STL with avoidance of the complexity of other interventions.

Despite its seemingly simple application, STL is not routinely performed in current clinical practice, mainly due to the lack of multiple high-quality RCTs demonstrating its efficacy and safety, which prevented guidelines from endorsing it as a possible therapeutic option. Moreover, the practical difficulties faced when conducting large-scale RCTs, accompanied by the uncertainty about long-term outcomes and the need for specialized training further hinder its widespread adoption.

### Strengths and limitations

To the best of our knowledge, this study is the first up-to-date systematic review that tackles such a critical topic with the rise of STL use in such a comprehensive manner. The only preceding review on the topic was not solely defined for STL intervention, and hence we aimed to be more focused on the present gap in literature. A thorough investigation was made with subgroup analysis in an attempt to address the limitations of our review, delineating possible underreported factors and estimating their contribution to the end result. One of the main limitations of our results was the small number of included studies comprising a relatively small sample size, which may lack the statistical power adequate to detect true differences in certain outcomes, particularly microvascular obstruction. Based on our PICO criteria, we excluded observational studies and included only randomized clinical trials in order to increase the power of our evidence, thus we included only four studies in our meta-analysis. Additionally, we could not assess the publication bias in our review because of the small number of included trials. Additionally, three of the four included studies did not perform a sham procedure in the control group, which may have introduced bias in the comparison between the two groups.

Furthermore, significant heterogeneity was reported in some outcomes, warranting caution in the interpretation of our findings. This heterogeneity could be attributed to variations in baseline characteristics such as comorbidities (e.g., diabetes mellitus, hypertension, and hyperlipidemia) and medications received by patients (e.g., aspirin, beta-blockers, statins, calcium channel blockers, and ACE inhibitors/ARBs). It may also be explained by the differences in baseline angiographic findings, including TIMI flow grade before PCI, the occluded vessel involved (LAD, LCx, or RCA), and the proportion of patients who underwent thrombus aspiration, predilatation, or postdilatation.

Additionally, Mathias et al. showed some concerns in the randomization process domain, which could introduce selection bias and influence internal validity. Since this study contributed to the infarction size outcome, its influence was evaluated by sensitivity analysis. The removal of this study altered the results, underscoring the potential bias impact.

Some additional limitations encountered include the lack of data on the duration of STL application, which has been previously suggested to influence the retrieved outcomes [47]. Another limitation lies in the assessment of STL intervention on a longer-term basis where mortality rate and quality of life can both be adequately assessed and hence support the development of more decisive guidelines. Our analysis included results pooled from short follow-up durations, which are insufficient to fully assess the long-term effects of STL. Finally, although ultrasound is widely used, its therapeutic parameters are still significantly overlooked, especially upon clinical trials documentation, hence limiting the generalizability of the conclusions.

### Implications for clinical practice

Our results endorse more detailed future research to provide adequate coverage on the topic of therapeutic application of ultrasound, specifically STL, with some points considered. Future RCTs should focus on larger sample sizes, standardized imaging protocols, and long-term follow-up in order to provide information about the effect of STL on survival and quality of life. Furthermore, a thorough life-time follow-up is highly recommended specifically in large-scale observational studies in order to delineate possible important outcomes such as mortality rate and long-term disabilities. Additionally, we recommend future RCTs to focus on different populations such as NSTEMI patients. We also advocate for future reviews on this topic that incorporate both interventional and observational studies. This approach would help expand the pool of included studies and enhance the overall sample size, providing a more comprehensive understanding of the subject. Some vital considerations for authorities should include more integration of modern modalities to help in accurate diagnosis of MVO incidents which could further assist in proper reporting of results and hence sufficient assessment of the intervention efficacy.

## Conclusion

In conclusion, our analysis reported that STL may show some improvements in certain cardiac functions evident by the improvement demonstrated in the LVEF post-infraction. Also, it could be a potential safe method to replicate given its low microvascular occlusion incidents when compared to other more intrusive interventions like PCI alone. However, due to the limited sample size, heterogeneity between the studies and the inherent limitations of the clinical trials included in our review, our results should be interpreted with caution and additional data based on RCTs with larger sample size and longer follow up period is required to fully assess the true value of STL and evaluate its clinical utility in the treatment of STEMI patients.

## Supplementary Information

Below is the link to the electronic supplementary material.

Supplementary Material 1

Supplementary Material 2

## Data Availability

The datasets used and/or analyzed during the current study are available from the corresponding author on reasonable request.

## Abbreviations

ACS: Acute Coronary Syndrome
STEMI: ST-Segment Elevation Myocardial Infarction
NSTEMI: Non-ST-Segment Elevation Myocardial Infarction
pPCI: Primary Percutaneous Coronary Intervention
MVO: Microvascular Obstruction
LVEF: Left Ventricular Ejection Fraction
EDV: End-Diastolic Volume
ESV: End-Systolic Volume
VLMI: Very Low Mechanical Index
MBs: Microbubbles
TPA: Tissue Plasminogen Activator
PRISMA: Preferred Reporting Items for Systematic Reviews and Meta-Analyses
OSF: Open Science Framework
RoB 2: Revised Cochrane Risk-of-Bias Tool for Randomized Trials
CI: Confidence Interval
MD: Mean Difference

## Code availability

No custom code or software application was developed for this study. Statistical analyses were performed using Review Manager (RevMan) version 5.4.1.
