# Supplementary material for: Efficacy of sonothrombolysis as an adjunct to primary percutaneous coronary intervention in ST-segment elevation myocardial infarction: a systematic review and meta-analysis
Source: J Thromb Thrombolysis. 2025 Sep 9;59(2):326–40. doi: 10.1007/s11239-025-03176-1 (PMC13018063; doi:10.1007/s11239-025-03176-1)

**(Supplementary Figure 1).** The bias-risk assessment diagram of the included articles**.** This figure demonestrates that all the included studies showed low risk if bias in all domains except Mathias et al., 2019 that showed some concerns in the randomization domain.


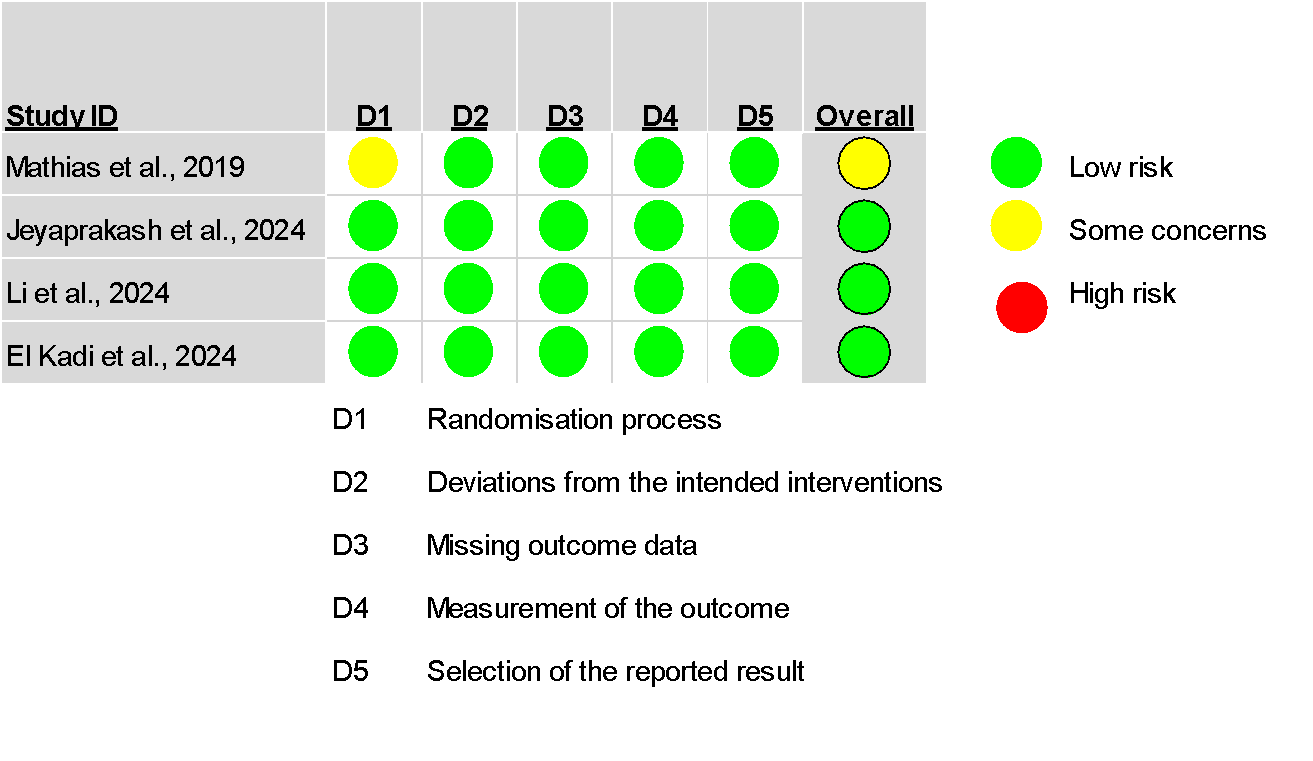


**(Supplementary Figure 2).** The bias evaluation bar graph of the included articles. All studies showed low risk of bias except Mathias et al., 2019 that showed some concerns in the final decision.


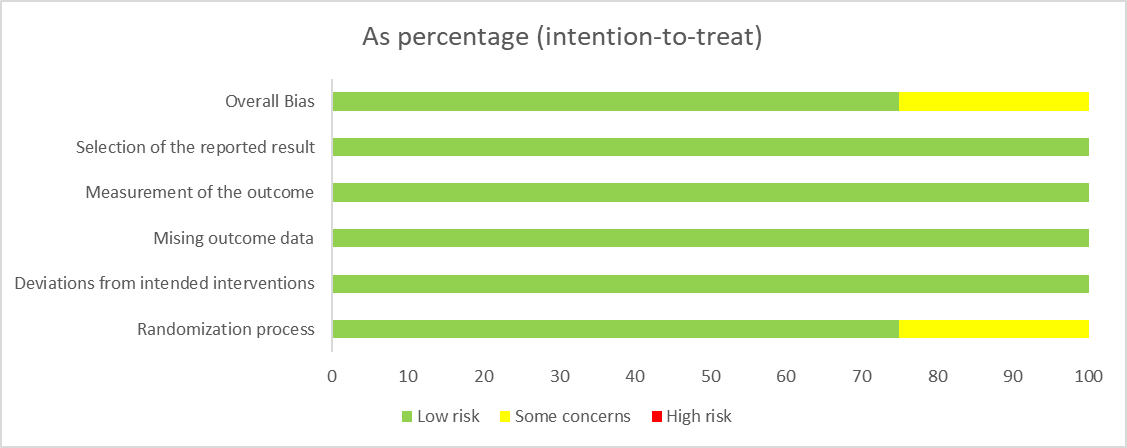


**(Supplementary Figure 3).** Left ventricular ejection fraction with leave-one out sensitivity analysis (After removing El Kadi 2024). The intervention group showed statistically significant improvement in both subgroups and in the overall pooled results (p < 0.05).

**
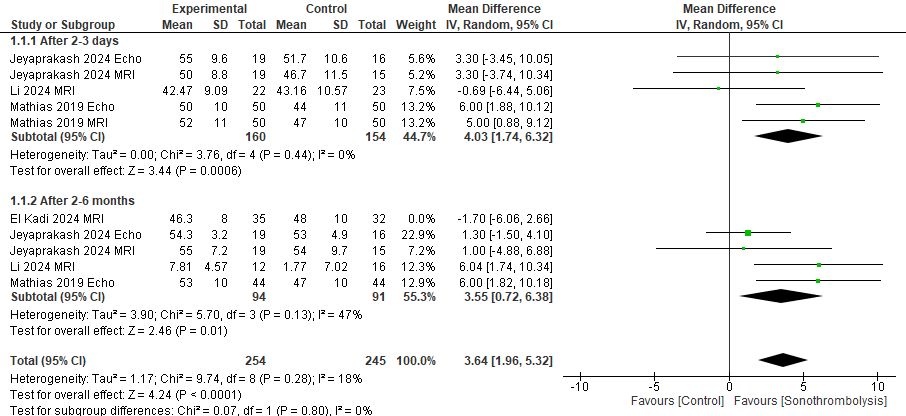
**

**(Supplementary Figure 4).** Infarction size with leave-one out sensitivity analysis (After removing Mathias 2019). No statistically significant difference was observed between the two interventions in either subgroup (p > 0.05)


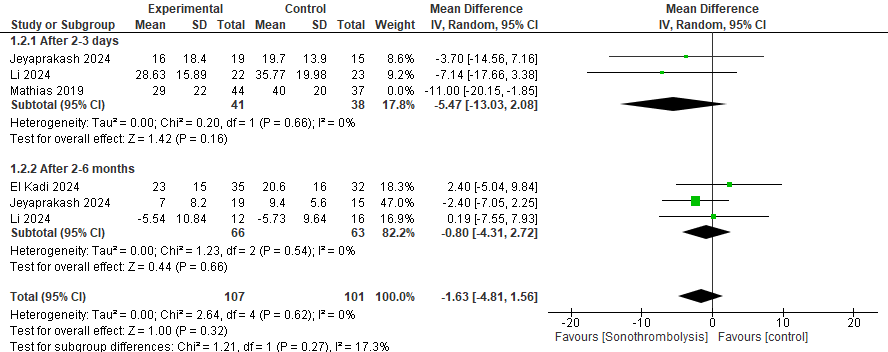

Supplement: Supplementary file 1 — Supplementary Material 1 [file 11239_2025_3176_MOESM1_ESM.docx]
