# Supplementary material for: Efficacy of sonothrombolysis as an adjunct to primary percutaneous coronary intervention in ST-segment elevation myocardial infarction: a systematic review and meta-analysis
Source: J Thromb Thrombolysis. 2025 Sep 9;59(2):326–40. doi: 10.1007/s11239-025-03176-1 (PMC13018063; doi:10.1007/s11239-025-03176-1)
Supplement: Supplementary file 2 — Supplementary Material 2 [file 11239_2025_3176_MOESM2_ESM.docx]

**(Supplementary Table 1).** Search strategy for different databases

| **Keywords** | **Database** |
| --- | --- |
| (“ultrasound-enhanced thrombolysis” OR “sonothrombolysis” OR “ultrasonics” OR “ultrasonic therapy”) AND (“Myocardial ischemia” OR “ischemic heart disease” OR Ischemia OR “Coronary Arteriosclerosis” OR “coronary artery disease” OR “Coronary Atherosclerosis” OR “Left Main Coronary Artery Disease” OR “Left Main Coronary Disease” OR “Left Main Disease” OR infarction OR “Myocardial Infarction” OR “heart attack” OR “Myocardial Infarcts” OR “Cardiovascular Stroke”). | ***PubMed*** |
|  | ***Scopus*** |
|  | ***Web of science*** |
|  | ***Cochrane library*** |
